# Study Protocol Titles: Erratum

**DOI:** 10.1097/MD.0000000000021984

**Published:** 2020-08-14

In recent issues, *Medicine* has published several PRISMA-P Systematic Review and Meta-Analysis Study Protocols^[1–7]^ that were not clearly labeled “a protocol for systematic review” in the article title.

The list of Study Protocols will be updated to include this subtitle in the article title.
